# Redox-regulation and life-history trade-offs: scavenging mitochondrial ROS improves growth in a wild bird

**DOI:** 10.1038/s41598-019-38535-5

**Published:** 2019-02-18

**Authors:** Alberto Velando, Jose C. Noguera, Alberto da Silva, Sin-Yeon Kim

**Affiliations:** Animal Ecology Group (GEA), Lab 97, Torre CACTI, Campus As Lagoas, Universidade de Vigo, Vigo, Spain

## Abstract

It has been proposed that animals usually restrain their growth because fast growth leads to an increased production of mitochondrial reactive oxygen species (mtROS), which can damage mitochondrial DNA and promote mitochondrial dysfunction. Here, we explicitly test whether this occurs in a wild bird by supplementing chicks with a mitochondria-targeted ROS scavenger, mitoubiquinone (mitoQ), and examining growth rates and mtDNA damage. In the yellow-legged gull *Larus michahellis*, mitoQ supplementation increased the early growth rate of chicks but did not reduce mtDNA damage. The level of mtDNA damage was negatively correlated with chick mass, but this relationship was not affected by the mitoQ treatment. We also found that chick growth was positively correlated with both mtDNA copy number and the mitochondrial enzymatic activity of citrate synthase, suggesting a link between mitochondrial content and growth. Additionally, we found that MitoQ supplementation increased mitochondrial content (in males), altered the relationship between mtDNA copy number and damage, and downregulated some transcriptional pathways related to cell rejuvenation, suggesting that scavenging mtROS during development enhanced growth rates but at the expense of cellular turnover. Our study confirms the central role of mitochondria modulating life-history trade-offs during development by other mechanisms than mtROS-inflicted damage.

## Introduction

In animals, early development is a critical life stage that strongly affects life-history trajectories and fitness^1^. Fast growth decreases the mortality risk at this stage^2^ and also allows earlier reproduction and higher competitive status, thereby potentially increasing lifetime reproductive success^3^. However, rapid growth may also be costly, for example by causing cellular ageing and damage^4,5^, although most of these costs are evident only during adulthood^6,7^. Selection has probably optimized growth rates through regulatory pathways, balancing short-term benefits with the delayed negative effects of cellular damage accumulation^8^. This may explain why organisms do not grow at their maximal rate^9^, but the exact mechanisms modulating this constraint on growth remain poorly understood.

Growth trajectory is extremely variable among individuals because it is a complex process that changes in response to nutrient availability and cellular homeostasis^10^. At the cellular level, mitochondria transduce most energy (acquired nutrients into ATP) in eukaryote cells by oxidizing nutrient components (i.e. oxidative phosphorylation, OxPhos), which is inevitably associated with the by-production of reactive oxygen species (ROS). When produced in excess, ROS cause oxidative damage of biomolecules^11,12^. Mitochondrial DNA (mtDNA) molecules are particularly susceptible to damage because they are located close to where mitochondrial ROS (mtROS) are produced and have limited protection and repair systems^13,14^. It has been suggested that mitochondrial dysfunction produced by oxidative mtDNA damage triggers an accelerated cycle of mtROS production and hence further damage (i.e. mitochondrial vicious cycle hypothesis^15^). Thus, fast growth may lead to an increased production of mtROS with damaging effects on mitochondrial DNA (mtDNA), which constrain the evolution of growth trajectories. Indeed, studies of vertebrates suggest that fast-growing individuals experience higher oxidative stress and oxidative damage^16–18^.

On the other hand, mtROS act as signalling molecules in the redox regulation of multiple cellular processes^19^, including pathways related to mitochondrial and/or cell proliferation and those preventing the accumulation of unrepaired damage. Thus, mtROS are involved in the regulation of mitochondrial biogenesis, i.e. the growth and division of mitochondria with increased mtDNA copies per cell^20^. During early development, high demands for increased energy supply are often accompanied by mitochondrial biogenesis^21^. In the Japanese quail (*Coturnix coturnix japonica*), for example, selection for a rapid growth increases the mitochondrial content per cell^22^. Elevated mtROS generation by damaged mitochondria also triggers the selective removal of damaged organelles (mitophagy) or even cells with excessive mtDNA damage^23^ to maintain systemic function^24^. Thus, cellular redox regulation may play a central role in the regulation of growth by altering the mitochondrial content and cell metabolism during early development.

Previous studies of vertebrates indicate that antioxidant supplementation increases growth rates, suggesting that oxidative stress constrains growth (reviewed in^25^). However, it is unclear whether antioxidants alleviate the damaging effects of mtROS or ROS of non-mitochondrial origin (e.g. NADPH oxidases) in developing animals, or alter the redox-regulation in the mitochondrial and cellular processes. Here, we explicitly tested the effect of mtROS on chick growth by supplementing a mitochondria-targeted ROS scavenger in a wild bird, the yellow-legged gull (*Larus michahellis*). We examined body mass changes during the first eight days of life, the period of most rapid growth in our study population. Growth differences during this period has long-lasting consequences in fledgling body mass^26^. We then explored physiological and molecular mechanisms, which potentially mediate the link between mitochondrial activity and chick growth. In this species, antioxidant supplementation (vitamin E) increases growth in some circumstances^27,28^, but it is unclear whether these results can be attributed to a mitigation of growth-related oxidative damage^28^.

We took advantage of a novel pharmacological product designed as a mitochondria-targeted antioxidant: the mitoubiquinone (mitoQ), a ROS-scavenging moiety linked to a lipophilic cation^29^. As a result of this covalent conjugation, mitoQ enters and several hundred-fold accumulates within mitochondria, where it selectively scavenges mtROS^30^. In this study, we examined the effect of mitoQ supplementation on chick growth, oxidative stress, mtDNA damage, mitochondrial density (mtDNA copy number and citrate synthase activity), and the expression of redox-regulated genes involved in mitochondrial biogenesis and cell turnover during early development. We studied these effects by using a non-invasive longitudinal sampling method (i.e. using blood cells). In vertebrates, blood cells are constantly regenerated from stem cells located in the bone marrow, with erythrocytes being by far the most common cell type. In birds, erythrocytes are fully functional cells with a nucleus, active transcription/translation (e.g.^31,32^) and functional mitochondria^33^. The respiratory profiles and mitochondrial abundance of avian erythrocytes provide surrogate information about an organism’s mitochondrial function^34^ and have been shown to correlate with species-specific lifestyle^35^. Thus, avian blood cells are suitable to examine mitochondrial metabolism and the expression of candidate genes^36^.

In this study, we first developed assays (primers and conditions) of quantitative polymerase chain reaction (qPCR) to estimate the relative number of mtDNA copies and measure mtDNA damage in the yellow-legged gulls, a non-model wild bird. We also developed assays to quantify the transcript levels of four redox-regulated nuclear genes involved in mitochondrial biogenesis and cell turnover, with possible consequences on growth patterns. In relation to mitochondrial proliferation, we examined the expression of the nuclear respiratory factor 1 (*NRF1*), a transcriptional factor that activates nuclear genes required for mtDNA replication^20^, and the expression of sirtuin 1 (*SIRT1*), a histone deacetylase that stimulates transcriptional pathways promoting mitochondrial biogenesis^37^. We also examined the transcriptional abundance of the caspase-7 (*CASP7*), a gene encoding a cysteine protease that regulates mitochondrial events involved in the cellular death machinery^38^; this gene is upregulated in avian blood cells after malaria infection^39^. Lastly, we analyzed the expression of proto-oncogene receptor tyrosine kinase (*KIT*), a redox-regulated gene involved in the turnover of blood cells^40^. Cell death and proliferation may have evolved to preserve functional cells and tissues by responding to elevated ROS generation in defective mitochondria^23^.

In our study, gull chicks supplemented with mitoQ are expected to enhance their early-life growth if mtROS constrain early development. According to the mitochondrial vicious cycle hypothesis, mtROS scavenging by mitoQ should reduce mtDNA damage, allowing enhanced mitochondrial activity. Mitochondrial biogenesis is triggered in response to cellular signals of energy demands, such as elevated ROS level^20^. Thus, mitoQ supplementation may alter the redox-regulation of cell mitochondrial turnover, reducing the mtDNA copy number and downregulating genes involved in mitochondrial biogenesis (*NRF1* and *SIRT1*). Since cellular turnover processes are activated by mtROS, we also expected a downregulation of the transcriptional pathways involved in cell death (*CASP7*) or cell development (*KIT*) in mitoQ-supplemented chicks.

## Results

One day after hatching, prior to supplementation, chicks assigned to the control and mitoQ supplement groups did not differ in mtDNA damage, mtDNA copy number, and body mass (P > 0.25 in all cases).

### MitoQ effects on body mass

**Figure 1:**
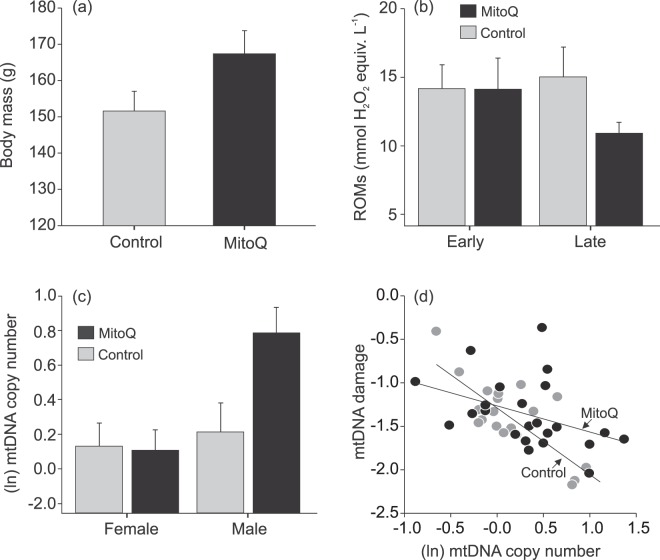
The effect of mitoQ supplementation in yellow-legged gull chicks at age eight days. (**a**) Average body mass according to mitoQ supplementation. (**b**) Plasma levels of reactive oxygen metabolites (ROMs) according to mitoQ supplementation and hatching date. For illustrative purposes only, hatching date was categorized in early and late according to median hatching date (3 June). (**c**) mtDNA copy number according to mitoQ supplementation and sex. (**d**) The relationship between mtDNA damage and copy number according to mitoQ supplementation.

MitoQ supplementation had a significant effect on chick body mass at age eight days (β_control_ = −0.53 ± 0.21; *P* = 0.020; Supplementary Table S1); the mitoQ-supplemented chicks were heavier than the controls (Fig. 1a). Additionally, early hatched chicks were heavier than late chicks (Supplementary Table S1), but neither the sex nor hatchling mass influenced chick body mass (Supplementary Table S1).

### MitoQ effects on oxidative stress and mitochondria

There was an interacting effect of mitoQ supplementation and hatching date on the ROMs level in plasma in 8-day-old chicks (*F*_1,20.3_ = 8.68; *P* = 0.008; Supplementary Table S2). MitoQ decreased ROMs in plasma but only in chicks hatching later in the season (Fig. 1b). Male chicks showed significantly higher ROMs level than female chicks (Supplementary Table S2). Plasma ROMs level was not significantly affected by hatching order (Supplementary Table S2).

The level of mtDNA damage in blood cells at age 8 days was not affected by mitoQ supplementation (β_control_ = −0.14 ± 0.31; *P* = 0.65; Supplementary Table S2) or any other variable included in the model (Supplementary Table S2). On the other hand, relative mtDNA copy number in blood cells was affected by the interaction between MitoQ supplementation and sex (*F*_1,36_ = 5.12; *P* = 0.030; Supplementary Table S2). MitoQ increased mtDNA copy number in male but not in female chicks (Fig. 1c). Hatching order and hatching date did not affect mtDNA copy number (Supplementary Table S2). The levels of mtDNA damage and copy number were negatively related, and the slope was steeper in the control chicks than in the mitoQ-supplemented chicks (mtDNA copy number x mitoQ, *F*_1,39_ = 5.40, *P* = 0.025). Thus, the mitoQ-supplemented chicks with high numbers of mtDNA copies showed increased mtDNA damage compared to the control chicks with similar numbers of mtDNA copies (Fig. 1d).

MitoQ supplementation did not affect the level of citrate synthase activity in blood cells (β_control_ = 0.11 ± 0.18; *F*_1,20.1_ = 0.38, *P* = 0.53), but this enzymatic activity was affected by chick order (β_first_ = −0.57 ± 0.19; *F*_1,21.5_ = 9.14, *P* = 0.006); second-hatched chicks showed a higher level of activity (17.65 ± 1.14 nmol min^−1^ mg^−1^ protein) than first chicks (14.55 ± 1.08 nmol min^−1^ mg^−1^ protein). Sex, hatching date and all two-way interactions did not affect citrate synthase activity (all *P* > 0.12). Importantly, the level of citrate synthase activity in blood cells correlated positively with mtDNA copy number (*r* = 0.31, *P* = 0.043) and negatively with mtDNA damage (*r* = −0.34, *P* = 0.024).

### Relationship between body mass growth and mitochondria

**Figure 2:**
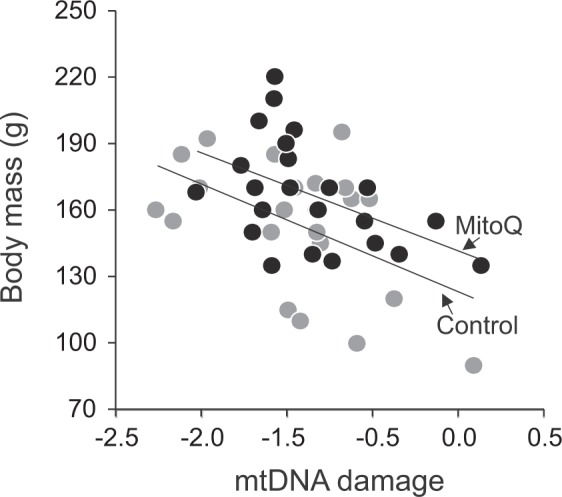
The relationship between body mass and mtDNA damage in yellow-legged gull chicks at age eight days.

The effect of the treatment on body mass remained significant when mtDNA damage, mtDNA copy number, or citrate synthase activity was included as a covariate in the analysis (Supplementary Table S3). Body mass was strongly and negatively related to the level of mtDNA damage (β = −0.34 ± 0.12, *P* = 0.009; Supplementary Table S3), and this relationship was similar in both treatment groups (mtDNA damage × mitoQ, *F*_1,32.7_ = 0.18, *P* = 0.67). Thus, chicks with more mtDNA damage were smaller in both treatment groups (Fig. 2). Similarly, chick body mass was positively related to mtDNA copy number (β = 0.28 ± 0.13, *P* = 0.043; Supplementary Table S3) and citrate synthase activity (β = 0.34 ± 0.13, P = 0.015; Supplementary Table S3) in both groups (two-way interactions *P* > 0.35).

### MitoQ effects on gene expression of candidate genes

**Figure 3:**
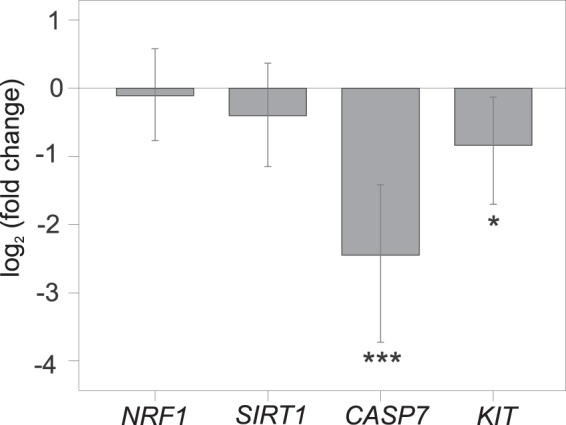
Effects of mitoQ supplementation on the expression (via real-time qPCR) of candidate genes in blood cells of eight-day-old chicks. Positive and negative values indicate up- and down-regulation, respectively, in mitoQ supplemented chicks. ***p < 0.001, *p < 0.05.

Analyses of mRNA levels indicate that genes involved in apoptosis and signalling pathways, *CASP7* and *KIT*, were downregulated in the mitoQ-supplemented chicks (Fig. 3; Supplementary Table S4). The expression of genes involved in mitochondrial metabolism, *NRF1*, and *SIRT1*, did not differ between the control and mitoQ chicks (Supplementary Table S4). Hatching order influenced gene expression; second-hatched chicks overexpressed *NRF1*, *SIRT1*, and *CASP7* genes compared to first-hatched chicks (Supplementary Table S4), suggesting a higher regulation of mitochondrial and cellular metabolism in second chicks.

## Discussion

This study suggests that scavenging mtROS during development can induce changes in mitochondrial and cellular processes with important consequences in growth rate. In our experiment, the mitoQ-supplemented chicks grew heavier than the control chicks independently of their level of mtDNA damage. MitoQ supplementation did not reduce mtDNA damage in blood cells, but increased mitochondrial content (in male chicks), altered the relationship between mtDNA abundance and damage, and affected the transcriptional pathways related to cell rejuvenation. Mitochondrial DNA copy number and damage had a positive and negative effect, respectively, on chick growth, highlighting the importance of mitochondrial abundance and quality during early development. Overall, our study confirms the central role of mitochondria modulating growth patterns during development.

MitoQ supplementation probably alleviated oxidative stress, as shown by the reduced level of plasma ROMs in the mitoQ-supplemented individuals, although this effect appeared only among late-hatched chicks. Nevertheless, contrary to our prediction, reducing mtROS did not alleviate mtDNA damage. We estimated mtDNA damage by qPCR, an assay designed to detect the general damage that blocks the progression of the DNA polymerase, such as DNA adducts and strand breaks. This assay is not specific to oxidative damage but sensitive to the damage produced by hydrogen peroxides^14,41^. Thus, our study is limited in that we did not measure direct oxidative damage in red blood cells, so we cannot explicitly evaluate the effect of mitoQ supplementation on oxidative stress. MitoQ might reduce ROS-induced damage, but this oxidative damage (if any) was not detected in our measure of global mtDNA damage. Recent evidence from model organisms suggests that ROS-induced damage may only represent a small fraction of the total mtDNA damage^42–44^.

We showed that the mtDNA copy number and the activity of citrate synthase of gull chicks were negatively related to mtDNA damage, suggesting a relationship among mtDNA damage and mitochondrial content and activity. Importantly, growth was correlated with these mitochondrial variables (negatively with mtDNA damage and positively with both mtDNA copy number and the activity of citrate synthase), confirming the link between mitochondrial performance and growth (e.g.^22^). These relationships were not affected by mitoQ supplementation, indicating that mtDNA damage and dysfunction may constrain growth rates by mechanisms other than ROS-inflicted damage. In our study, mitoQ supplementation increased growth in gull chicks but without apparent effects on mtDNA damage. MitoQ may have principally functioned to allow the experimental chicks to increase their growth while maintaining mtDNA damage at a comparable level to the control chicks.

Several (non-exclusive) redox mechanisms may possibly explain how the mitoQ-supplemented chicks accelerated growth. ROS-scavenging might allow the chicks to increase mitochondrial efficiency, i.e. high ATP synthesis per unit of food consumed, which depends on the electrochemical potential^45^. Uncoupling proteins, located in the mitochondrial inner membrane, are able to dissipate the proton gradient^46^; these proteins are activated under high levels of mtROS^47,48^. In our study, mtROS scavenging by mitoQ may have contributed to reducing the activity of uncoupling proteins^47^ and hence increasing the efficiency of ATP production. Some empirical studies of birds support the positive relationship between fast growth and mitochondrial efficiency^49^, but not others^50^. Other possibility is that mitoQ increased mitochondrial content and hence the metabolic capacity^22^, but we only found evidence of this in males. The mitoQ-supplemented male chicks, but not females, showed increased mtDNA copy number. Male chicks also showed elevated levels of plasma ROMs compared to females, which suggest that the effects of ROS scavenging on mitochondrial content may depend on the cellular redox state. The elevated mtDNA content in the mitoQ-supplemented male chicks cannot be attributed to the upregulation of genes involved in mitochondrial biogenesis (*SIRT1* and *NRF1*), but may arise by mito-Q inhibition of mitophagy^51^. Independently of the mechanism, our study highlights the role of mitochondria in modulating sex-specific trade-offs during development.

Our experimental results also suggest that mitoQ administration affected a range of metabolic pathways involved in cell turnover through the regulation of gene expression levels. We found that the expression of *CASP7* was downregulated in the mitoQ-supplemented chicks. In birds, caspase-mediated cell death occurs mainly in young erythrocytes, and this pathway is mainly inactive in old bird erythrocytes (see^52^). Thus, the low abundance of caspase transcripts in blood cells of the mitoQ-supplemented chicks may indicate not only a reduction in apoptotic cell clearance^38^ but also a high abundance of old blood cells. Blood cells of the mitoQ chicks also showed a downregulation of the *KIT* gene, which encodes a cell surface receptor kinase that has a critical role in the proliferation and development of blood cells^53^. Interestingly, mitoQ supplementation affected the relationship between mtDNA abundance and damage, with the mitoQ chicks showing higher levels of damage than the controls when mtDNA was abundant. This effect may indicate the reduced proportion of young (undamaged) cells and/or differences in mitochondrial metabolism in the mitoQ chicks (see above). Downregulation of costly cellular processes, such as cell turnover and development, could also increase the amount of resources available for growth in the mitoQ-supplemented chicks. More studies are needed to establish the cost of these processes (if any).

On the other hand, it is interesting to note the effect of the competitive environment (hatching order) on gene expression patterns in blood cells of gull chicks. In yellow-legged gulls, the first two chicks typically hatch with only one day of difference and they are strong competitors for parental care. Second chicks grow faster and show different behavioural strategies from their senior broodmates^17,54^. In this study, second-hatched chicks showed higher levels of mitochondrial activity (citrate synthase) and transcript abundance of genes related to cellular death (*CASP7*) and mitochondrial biogenesis (*SIRT1, NRF1*). Thus, these gene expression patterns suggest a higher mitochondrial and cell turnover in second-hatched chicks, probably as a response to their faster metabolism.

Here, we provide evidence that mitochondria play a key role during the period of rapid growth. We found that chick growth was affected not only by the level of mtROS but also by mitochondrial damage and content. ROS generated by mitochondria may act as a signal that triggers cellular and somatic protection against diverse molecular damage (not only and even not primarily from oxidative stress^55,56^). In animals, the cost of fast growth may arise due to increased efficiency of ATP-dependent processes^45^ at the expense of cell rejuvenation and selection acting on the regulatory pathways to optimize this trade-off. A more complex scenario may arise if molecular signals are also costly to produce (e.g. in terms of oxidative injury). Future studies should explore the long-term consequences of mtROS scavenging during growth on somatic maintenance to fully understand how growth rates are optimized through regulatory pathways.

In conclusion, our results suggest that elevated mtROS and impaired mitochondria additively constrain body mass growth rate in wild animals. Our study provides evidence that mitochondria may govern life-history trade-offs by additional mechanisms other than oxidative damage. We propose that selection may have shaped mitochondrial redox-regulation as a quality control mechanism to maximize cellular functions (e.g. mitochondrial efficiency), while preventing delayed detrimental effects produced by accumulated damage. Quality control systems may translate internal states into molecular signals linking life-history traits separated in time, and hence not subject to a direct allocation trade-off^4,7^. Although the study of mitochondrial function is challenging, new tools have been recently developed in non-model organisms (e.g.^34,57^) and here we additionally provide some molecular tools to analyse mitochondrial density and damage. Combining multiple measures of mitochondrial efficiency, metabolism and integrity with redox-regulated processes, affecting the rate of living, has the potential to disentangle the complex role of mitochondria in shaping growth trajectories and more general life-history trajectories^58^.

## Methods

The field experiment was carried out from April to June 2016 in a colony of yellow-legged gulls in Sálvora Island, Spain. Yellow-legged gulls lay eggs at 1- to 3-days interval to complete a clutch of three eggs (modal clutch size). The study area was surveyed once daily during egg laying. Nests were marked with numbered sticks and eggs were marked for identification of laying order. We used 30 nests containing a clutch of three eggs with known laying date. Prior to hatching, we installed a fenced enclosure around each nest to keep chicks in their territory^27^. Hatchlings were individually marked by using a leg flag. In three nests, all eggs failed to hatch.

Ethical considerations were taken into account in the experiment design to reduce the sample size as low as possible and avoid any potential damage caused by mitoQ supplementation in our wild birds, while still eliciting a measurable response. The field study was carried out under a permission from the Parque Nacional das Illas Atlánticas and Xunta de Galicia (364/RX598377), and all field procedures complied with the current laws of Spain (RD53/2013). The experimental protocol was approved by the Committee of the Ethics of Animal Welfare of the University of Vigo (08/2015).

We allocated control and experimental chicks within the same brood, reducing between brood differences in parental care. In this population, the first two chicks experience similar nutritional conditions, but the third suffers a competitive disadvantage^27^. Thus, within a brood, first- and second-hatched chicks were randomly assigned to either a non-supplement (*n* = 27 chicks) or a mitoQ supplement group (*n* = 27 chicks). Chicks assigned to the mitoQ group received a daily dose of 0.3 mg mitoQ (mitoquinone mesylate, Chemistry Department, University of Otago) in 0.5 ml water during five consecutive days via oral administration, beginning one day after hatching (day 1). This dose (c. 2–3 mg·kg^−1^) was well below the maximum tolerated dose in mice (c. 20 mg·kg^−1^ ^59^). Current evidence suggests that mitoQ has no deleterious effects *in vivo*, even when it is long-term administered at high dosages^60,61^. In a pilot study, we did not observe any detrimental effect of this dosage during four days (*n* = 5 chicks), and during our experiment, mitoQ supplemented chicks increased their performance without any apparent negative effects (see Results). The control group received the same amount of water without mitoQ. We did not use the lipophilic cation (Triphenylphosphonium, TPP) as control group because this moiety alone (i.e. lacking the antioxidant group) impairs mitochondrial membrane potential and respiratory chain activity, and indeed its use in humans is discouraged due to its potentially harmful effects^62^.

We weighed and blood sampled the chicks at age one (immediately before the first supplementation) and eight days. In our study population, body mass at eight days of age is a good proxy of final body mass at the end of the growing period (30 days of age; *r* = 0.52, *n* = 133 *P* < 0.001, our unpublished data). Blood samples were collected from the brachial vein using a sterile needle and heparinized capillary tubes and kept cold until stored in liquid nitrogen after separation of plasma and red blood cells (within a few hours after collection; approx. 6 min × 4000 g). A blood aliquot (c. 50 μl) at age eight days was mixed (1:5) with RNA*later* (Ambion) for analysis of gene expression and stored in liquid nitrogen. From blood cells, DNA was extracted by using DNeasy Blood and tissue kit and following the manufacturer’s instructions (Qiagen). The quantity and purity of the genomic DNA were measured using Take 3 on a Synergy microplate reader spectrophotometer (BioTek). In one sample from a one-day-old chick, the amount of DNA extracted was too low to perform all the analyses. Sex was determined by molecular markers.

### Reactive oxygen metabolites (ROMs)

We estimated the level of reactive oxygen metabolites (ROMs) in plasma, a possible indirect proxy of ROS production in the whole organism (see Supplementary Methods). Briefly, reaction of ROMs in plasma (5 μL) with N,Ndiethyl-p-phenylenediamine was spectrophotometrically measured and expressed as mmol H_2_O_2_ equivalent·L^−1^ (see Supplementary Methods). ROMs levels were not affected by plasma triglycerides (ESM).

### Mitochondrial DNA copy number

We estimated relative mtDNA copy number in blood cells by measuring the amount of mitochondrial DNA relative to the nuclear DNA by real-time qPCR. We used glyceraldehyde-3-phosphate dehydrogenase (*GAPDH*) and cytochrome oxidase subunit 1 (*COI*) as the unique single copies in the nuclear and mitochondrial genome (see details of design and validation of primers and qPCR conditions in Supplementary Methods and Table S5). Briefly, the assays were performed in a total volume of 25 µl on a StepOnePlus (Applied Biosystems). *COI* and *GAPDH* reactions were performed on separate plates (see details in Supplementary Methods). The relative mtDNA copy number was transformed by natural logarithm prior to data analyses.

### Mitochondrial DNA damage

We estimated mtDNA damage using a quantitative ‘long’ PCR-based assay based on the principle that DNA damage slows down or block DNA polymerase advance^41^. This assay has been previously validated in several species (see^63^). The levels of lesions were quantified by the amplification of large mitochondrial genomic fragment and normalized by a short mitochondrial fragment (*COI* gene), which is less likely to be affected by the random damage (see details of design and validation of primers and PCR conditions in Supplementary Methods and Table S5). qPCRs were performed in SureCycler 8800 thermal cycler (Agilent) using Herculase II fusion DNA polymerase (Agilent) and DNA was quantified using PicoGreen (dsDNA assay kit Invitrogen) in a Synergy HT BioTek microplate reader (see details in Supplementary Methods). Relative DNA lesion frequencies were normalized to reference as described by Furda *et al*.^64^. Briefly, we estimated the relative damage per DNA strand as the ratio of fluorescence values of large and small mtDNA target in each sample (RS) and in the reference (RR). Normalized mtDNA damage was determined as –ln(RS/RR).

### Citrate synthase activity

Citrate synthase activity was measured in blood samples taken at age eight days. Citrate synthase activity is a good proxy of the abundance of functional mitochondria (see Supplementary Methods). We followed the assays previously described in Spinazzi *et al*.^65^ with minor modifications. Briefly, blood cells homogenate was reacted with oxaloacetic acid and the change in absorbance was monitored for 3 min. The citrate synthase activity was expressed as the rate of production of thionitrobenzoic acid (nmol min^−1^ mg^−1^ of protein; see details in Supplementary Methods).

### Gene expression

The expression profiles of candidate genes were estimated based on relative quantification of mRNA transcripts, assayed by RT-qPCR using a StepOnePlus Real-Time PCR Systems (Applied Biosystems). Total RNA was isolated from blood samples in RNAlater from eight-day-old chicks and first-strand cDNAs were synthesized with qScript cDNA Synthesis Kit (Quanta Biosciences). Some samples (n = 14) showed low amounts of RNA and were excluded from qPCR analyses. Further details on RNA isolation and cDNA synthesis can be found in Supplementary Methods.

Beta-actin gene (*ACTB*) was used as reference gene^66^, and gene-specific primers were designed using sequence information obtained from previously published sequences for *ACTB*, *KIT*, *CASP7*, and *SIRT1* genes in the yellow-legged gull^67^ or using the available sequences in Charadriiformes (see Supplementary Methods and Table S5). The level of expression was measured in a 20 µl reaction volume, and all reactions were performed in duplicate (see details in Supplementary Methods).

### Sample size and statistical analyses

We first analyzed whether initial values in mtDNA damage, mtDNA copy number, and body mass, prior to the experiment, differed between experimental groups using linear mixed models (LMMs) including the mitoQ treatment (mitoQ supplement and control), chick order (first and second), sex and hatching date (continuous variable; from 30 May to 5 June, median 3 June) as fixed terms and nest identity as a random term.

Among 54 hatchlings, 45 chicks (22 control and 23 mitoQ chicks) survived until age eight days. In mtDNA copy number analysis, one sample failed to produce accurate amplification (see above). To facilitate the interpretation of the main effects, we used mean-centered (z-score) continuous covariates^68^. The effects of mitoQ supplementation on ROMs, mtDNA damage, mtDNA copy number and citrate synthase activity of chicks at day eight were analyzed using LMMs, including treatment, chick order and sex as fixed factors, hatching date (continuous variable) as a covariate and nest identity as a random term. Initial values (one day after hatching) of mtDNA damage and mtDNA copy number were also included as covariates in their respective analyses. Note that citrate synthase activity at one day of age was not quantified due to insufficient volume of blood samples. We also tested the interactions between treatment and all fixed terms (including initial values), but only significant interactions were retained in the models. Additionally, we also tested if the relationship between mtDNA variables varied between treatments by running a LMM of mtDNA damage including mtDNA copy number, treatment and its interaction as fixed effects, initial mtDNA damage as a covariate and nest identity as a random term. We calculated the coefficients of determination for mixed models indicating the proportion of variance explained by the fixed factors only (marginal, R^2^_LMM(m)_) and by both the fixed and random effects (conditional, R^2^_LMM(c)_)^69^.

We analyzed relative gene expression in a subsample (see above) of chicks at age eight days (*n* = 31, 14 control and 17 mitoQ chicks) in a multivariate generalized linear mixed model with a Poisson-lognormal distribution and a Bayesian Markov Chain Monte Carlo (MCMC) sampling scheme by using the MCMC.qpcr package^70^ implemented in R. Poisson-lognormal distribution is particularly suitable to analyze RT-qPCR transcript data because low-abundant targets typically show high variance. In the model, hatching order and treatment were included as fixed factors. The effect of sex was not significant for any gene, so it was removed from the analysis to avoid overparameterization in the calculation of multivariate coefficients. Variation in the quality and quantity of biological material among samples (global effects) and inter-run variation were taken into account in the model by including sample and plate as random effects. Nest identity was also included as an additional random effect. The model included ACTB as reference gene (‘soft normalization’ in MCMC.qpcr package). The model satisfied the linearity and homoscedasticity criteria, as indicated by diagnostic plots. The statistical significance of posterior distribution of parameters was estimated as a Bayesian two-sided *p*-value (pMCMC), which is twice the fraction of all sampled values by MCMC that crosses zero with respect to the mean.

The effect of the treatment on body mass was also analyzed in a LMM including treatment, chick order and sex as fixed factors, hatching date and chick body mass at age one as covariates and nest identity as a random term. All interactions between treatment and other fixed terms were non-significant, so they were removed from the model. Interacting effects of treatment with mtDNA damage, copy number and citrate synthase activity on body mass were analyzed in three separate additional models. Results are presented as means ± standard error, and the significance level was set at P < 0.05.

## Supplementary information

Supplementary Material

## Data Availability

Data reported in this paper have been deposited in the Figshare digital repository, https://figshare.com/s/537a7b803166fe45f0ae.
