# Supplementary material for: Redox-regulation and life-history trade-offs: scavenging mitochondrial ROS improves growth in a wild bird
Source: Sci Rep. 2019 Feb 18;9:2203. doi: 10.1038/s41598-019-38535-5 (PMC6379414; doi:10.1038/s41598-019-38535-5)
Supplement: Supplementary file 1 — Supplementary Material [file 41598_2019_38535_MOESM1_ESM.pdf]

Redox-regulation and life-history trade-offs: Scavenging mitochondrial ROS improves growth in a wild bird

Alberto Velando, Jose C. Noguera, Alberto da Silva, Sin-Yeon Kim

SUPPLEMENTARY TABLES S1-S5

Table S1. Summary of the linear mixed model testing the effect of mitoQ supplementation on body mass in yellow-legged gull chicks at age eight days. Interactions between fixed factors were not significant.

Fixed effects	β (*se*)	*F* (*df*)	*P*
MitoQ (control)	-0.53 (0.21)	6.58 (1,17)	**0.020**
Sex (female)	-0.40 (0.24)	2.72 (1,24)	0.112
Hatching order (first)	0.34 (0.21)	2.79 (1,17.7)	0.112
Hatching date	-0.38 (0.16)	5.41 (1,24.6)	**0.028**
Body mass day 1	0.13 (0.14)	0.86 (1,37.8)	0.360
Random effects	*Variance (se)*	*Wald Z*	*P*
Nest ID	0.35 (0.22)	1.58	0.114
Residual	0.42 (0.16)		
	$R^2_{LMM(m)}$ = 0.236		
	$R^2_{LMM(c)}$ = 0.587		

Table S2. Summary of the linear mixed models testing the effects of mitoQ supplementation on ROMs level in plasma and mtDNA damage and mtDNA copy number in blood cells in yellow-legged gull chicks at age eight days. Only significant interactions were retained in the models

	ROMs			mtDNA damage			mtDNA copy number		
Fixed effects	β (*se*)	*F* (*df*)	*P*	β (*se*)	*F* (*df*)	*P*	β (*se*)	*F* (*df*)	*P*
MitoQ (control)	-0.18(0.20)	0.77 (1,15.2)	0.392	-0.14 (0.31)	0.21 (1,38)	0.650	-1.28 (0.50)	3.97 (1,36)	0.054
Sex (female)	-0.73 (0.26)	8.24 (1,21.8)	**0.009**	0.37 (0.33)	1.29 (1,38)	0.264	-1.34 (0.46)	4.27 (1,36)	**0.046**
Hatching order (first)	0.03 (0.21)	0.18 (1,17.4)	0.896	0.15 (0.31)	0.24 (1,38)	0.624	0.04 (0.28)	0.02 (1,36)	0.884
Hatching date	-0.40 (0.21)	0.09 (1,29.0)	0.760	0.26 (0.17)	2.13 (1,38)	0.153	-0.28 (0.16)	3.29 (1,36)	0.078
Initial value[1]	-0.02 (0.13)	0.03 (1,32.6)	0.855	-0.11 (0.16)	0.49 (1,38)	0.488	0.25 (0.16)	2.39 (1,36)	0.131
MitoQ x Hatching date	0.70 (0.24)	8.68 (1,20.3)	**0.008**	-	-	-	-	-	-
MitoQ x Sex	-	-	-	-	-	-	1.36 (0.60)	5.12 (1,36)	**0.030**
Random effects	*Variance (se)*	*Z*	*P*	*Variance (se)*	*Z*	*P*	*Variance (se)*	*Z*	*P*
Nest ID	0.35 (0.23)	1.53	0.120	0	-	-	0	-	-
Residual	0.44 (0.17)			1.02 (0.23)			0.79 (0.18)		
	$R^2_{LMM(m)}$ = 0.172			$R^2_{LMM(m)}$ = 0.102			$R^2_{LMM(m)}$ = 0.303		
	$R^2_{LMM(c)}$ = 0.661			$R^2_{LMM(c)}$ = 0.102			$R^2_{LMM(c)}$ = 0.303		

[1]ROMs, mtDNA copy number and mtDNA damage at one day of age, in their respective analyses.

Table S3. Summary of the linear mixed models for the effect of mitoQ supplementation on chick body mass at age eight days, including mtDNA damage, mtDNA copy number or citrate synthase activity as a covariate in the model.

	including mtDNA damage			*including mtDNA copy number*			*including citrate synthase activity*		
Fixed effects	β (*se*)	*F* (*df*)	*P*	β (*se*)	*F* (*df*)	*P*	β (*se*)	*F* (*df*)	*P*
MitoQ (control)	-0.63 (0.21)	8.43 (1,18.7)	**0.009**	-0.52(0.23)	5.21 (1,15.6)	**0.037**	-0.62 (0.23)	7.50 (1,15.2)	**0.015**
Sex (female)	-0.40 (0.24)	2.67 (1, 27.9)	0.113	-0.25(0.27)	0.88 (1,21.4)	0.359	-0.46 (0.25)	3.39 (1,28.6)	0.076
Hatching order (first)	0.41 (0.22)	3.64 (1,18.4)	0.072	0.35 (0.22)	2.79 (1,15.6)	0.138	0.53 (0.28)	5.05 (1,18.6)	**0.037**
Hatching date	-0.32 (0.15)	4.774 (1,25.8)	**0.038**	-0.42 (0.15)	8.18 (1,21.6)	**0.009**	-0.35 (0.14)	5.90 (1,18.34)	**0.026**
Body mass day 1	0.12 (0.13)	0.85 (1,32.5)	0.362	0.17 (0.13)	1.69 (1,32.3)	0.203	0.17 (0.13)	1.80 (1,28.8)	0.191
mtDNA damage	-0.34 (0.12)	7.61 (1, 34.9)	**0.009**	-	-	-	-	-	-
mtDNA copy number	-	-	-	0.28 (0.13)	4.41 (1,36)	**0.043**	-	-	-
citrate synthase activity	-	-	-	-	-	-	0.35 (0.13)	6.94 (1,23.8)	**0.015**
Random effects	*Variance (se)*	*Z*	*P*	*Variance (se)*	*Z*	*P*	*Variance (se)*	*Z*	*P*
Nest ID	0.15 (0.18)	0.83	0.402	0.16 (0.21)	0.75	0.452	0.10 (0.22)	0.44	0.661
Residual	0.48 (0.18)			0.50 (0.21)			0.54 (0.23)		
	$R^2_{LMM(m)}$ = 0.389			$R^2_{LMM(m)}$ = 0.374			$R^2_{LMM(m)}$ = 0.394		
	$R^2_{LMM(c)}$ = 0.535			$R^2_{LMM(c)}$ = 0.526			$R^2_{LMM(c)}$ = 0.487		

Table S4. Results from multivariate MCMC GLMMs testing the effects of MitoQ treatment on the expression of candidate genes in blood cells. Estimated coefficients expressed as natural logarithms of fold-changes, 95% credible intervals and *P*-values derived from the posterior distribution (P_{MCMC}) are provided.

Source of variation	Gene	estimate	lower 95% CI	upper 95% CI	p_{MCMC}
MitoQ (supplemented)	*CASP7*	-1.695	-2.582	-0.981	**<0.001**
	KIT	-0.580	-1.181	-0.090	**0.036**
	NRF1	-0.076	-0.533	0.401	0.750
	SIRT1	-0.279	-0.797	0.254	0.302
Hatching order (second)	*CASP7*	2.603	1.716	3.541	**<0.001**
	KIT	0.190	-0.363	0.672	0.458
	NRF1	0.553	0.048	1.021	**0.030**
	SIRT1	0.558	0.005	1.074	**0.044**

Table S5. Primers used for assays of qPCR. Tm, melting temperature

Gene	Sequence (5'-3')<br>Forward (F) Reverse (R)	Tm<br>(°C, F/R)	Amplicon size
Nuclear			
GAPDH	F: GGAGGGTGTATGGAATTCTTCCT<br>R: CACAACCGAACCACTCAGCTT	(65.1, 66.4)	69 bp
Mitochondrial			
COI	F: CCCTCTCACAATATCAAACCC<br>R: GGATCGAAGAATGTTGTGTTTAG	(62.1, 61.3)	142 bp
Large fragment	F: GCATCATGATTTAGCGAGAACAACC<br>R: AGAAGTAGGGGTGGAATGGGATTTT	(68.3, 68)	13 Kbp
Gene expression			
CASP7	F: CCTGTATCCTCCTAAGCCAC<br>R: CAGAGTCTCATTTGCAGGTC	(60.2,60)	205 bp
KIT	F: CAGAAACAGCCGTTTACGAG<br>R: GGTAACATCCTGATCGGTGTAG	(62.4,62.1)	163 bp
NRF1	F: CGTCGCACAAGTCAATTATTC<br>R: CCTGCGTCGTCTGGATAGTC	(62.1,64.8)	98 bp
SIRT1	F: TTGTTCAGCAGCATCTCATG<br>R: TGTCATATCATCCAGTTCAGG	(62.7,60)	98 bp
ACTB	F: GAAATTGTGCGTGACATCAAG<br>R: GGACTCCATACCCAAGAAAGATG	(61.2, 63.5)	194 bp

SUPPLEMENTARY METHODS: Laboratory analyses

Reactive oxygen metabolites (ROMs)

We estimated the level of reactive oxygen metabolites (ROMs) in plasma using the method described in[1]. In gull chicks, high levels of plasma ROMs are associated with stressful growing conditions[2]. This assay mainly measures secondary metabolites, as the alkoxy and peroxy radicals derived from the hydroperoxides formed by ROS attack on free unsaturated fatty acids[3], so a possible indirect proxy of ROS production Briefly, ROMs in plasma (5µL) were reacted with N,Ndiethyl-p-phenylenediamine to produce a coloured complex that can be measured spectrophotometrically at 495 nm (SynergyTM 2 Multi-Mode Microplate Reader, Bio-Tek Instruments, Inc.) and expressed as mmol H_2O_2 equivalent·L^{-1}. Samples were measured in duplicate (intra-class correlation coefficient, r_{ICCC} = 0.943, P < 0.001). We also analyzed plasma triglycerides (in a single measure; in a previous study on gull chicks[4], this assay showed high repeatability; r_{ICCC} = 0.96, P<0.001) with commercially available kits (Biosystems, Barcelona) to test any possible effect of recent food (lipid) intake on ROMs measures. However, triglycerides and ROMs levels in plasma were not correlated (r=0.019, P=0.907), so this measure was not taken into account in the statistical analysis.

Mitochondrial DNA copy number

We estimated relative mtDNA copy number measuring the amount of mitochondrial DNA relative to the nuclear DNA by real-time PCR on a StepOnePlus (Applied Biosystems). We used glyceraldehyde-3-phosphate dehydrogenase (*GAPDH*) as the unique single copy region in the nuclear genome, with primers (see Supplementary Table S5) previously validated for our study species[4]. We selected cytochrome oxidase subunit 1 (*COI*) gene as mitochondrial gene and primer sequences were designed to amplify a small fragment of *COI* gene using available sequences of ten gull species from GeneBank (Accession Numbers [AN]: AY666375.1; EU727229.1; GU571453.1; GU571451.1; GU571942.1; GU571944.1; GU571946.1; HM033522.1; HM033524.1; JF498790.1; JF499143.1). The amplification product was sequenced in a 3130 Genetic Analyzer (Applied Biosystems), and its identity confirmed with BLASTN (all significant alignments were produced by *Larus COI* gene). Additionally, a BLASTN of the sequenced product into the available bird genomes (including a Charadriiformes species, *Charadrius vociferous*) did not produce any significant result in nuclear genes. Melt curve and electrophoresis analyses also confirmed that a single amplicon was generated by PCR with the designed *COI* primers.

For the real-time PCR assay, the reactions were performed in a total volume of 25 µl including 9.6 ng of template DNA, primers at a final concentration of 500 nM and 12.5 µl Luminaris Color HiGreen High ROX qPCR Master Mix (Thermo Scientific). *COI* and *GAPDH* reactions were performed on separate plates; the qPCR conditions for COI were: 10 min at 95°C, followed by 40 cycles of 15 s at 95°C, 60 s at 60°C; conditions for *GAPDH* were: 10 min at 95°C, followed by 40 cycles of 15 s at 95°C, 30 s at 58°C and 30 s at 72°C. An individual sample was used as a reference sample, which was included in triplicate on every plate. Triplicates of each sample were always allocated on the same plate, and similar numbers of samples from different experimental groups were included in each plate. Quantification cycle (hereafter Cq) values were highly repeatable in both amplicons (*COI*, r_{ICCC}=0.99, p < 0.001; *GAPDH,* r_{ICCC}=0.96, p < 0.001). The reference sample was used to calculate both the within- and among-plate variations. Mean intra-assay and inter-assay variations (CV) of the Cq values were <1% in all cases. An amplification outlier was excluded from the analyses. The efficiency of each amplicon was estimated from the slopes of the amplification curves for each qPCR reaction and averaged for each gene using LinRegPCR software[6]. The mean reaction efficiencies were 1.880 (range 1.876–1.883) for COI and 1.887 (range 1.886–1.889) for GAPDH. The relative mtDNA copy number of each sample was calculated as $(Ef_{COI})^{\Delta CqCOI}/(Ef_{GAPDH})^{\Delta CqGAPDH}$. Here, *Ef* is the amplicon efficiency, and ΔCq the difference in Cq-values between the reference sample and the focal sample. We used the mean Cq values of the triplicates in all calculations. The relative mtDNA copy number was transformed by natural logarithm prior to analyses. Final values of mtDNA copy number were highly repeatable when estimated for each replicate (r_{ICCC}= 0.991, P < 0.001). Importantly, relative mtDNA copy number was significantly correlated with citrate synthase activity (see results), a proxy of mitochondrial density.

Mitochondrial DNA damage

We estimated mtDNA damage using a quantitative 'long' PCR-based assay based on the principle that DNA damage slows down or block DNA polymerase advance[7-9]. Thus, the levels of lesions were quantified by the amplification of large genomic fragment and normalized by a short fragment, which is less likely to be affected by the random damage. This assay has been previously validated in several species to detect mitochondrial DNA damage induced by a large number of damaging agents (see[10]). For DNA quantification, we used the PicoGreen dsDNA assay kit Invitrogen) and a Synergy HT BioTek microplate reader (excitation wavelength 480 nm, emission wavelength 520 nm).

We designed primers to amplify a large mtDNA target (almost the entire mitochondrial genome, but excluding the D-loop to avoid bias from this mutation hotspot) by using the known complete mitochondrial genomes of five gull species (AN: AY293619.1; JX155863.1; KJ507782.1; KM507782.1; KT943749.1). We optimized the primer combination (Supplementary Table S5) and PCR conditions to produce a bright and unique band of predicted amplicon size (13 kbp) without any secondary product. We also selected four digestion enzymes (ApaI, Eco52I, SacII XBaI, Thermo Scientific) that produce a consistent digestion pattern (number and size of digested fragments) in the expected mitochondrial fragment across the five gull species used to design the primers (see above). Thus, the identity of the product amplified by the designed primers in our study species was confirmed by restriction digestion with these enzymes and gel electrophoresis of cut products. We used the primers for COI gene to amplify a short mtDNA target (see above; Supplementary Table S5).

qPCRs were performed in SureCycler 8800 thermal cycler (Agilent) using Herculase II fusion DNA polymerase (Agilent). PCR conditions were optimized for each amplicon (short and large mtDNA target) to keep amplification in the exponential phase[11]. Reaction mixtures contained 0.25 µM primers, 0.5 µL Dimethylsulfoxide (2%), 25 mM of each dNTP, 5 µL Herculase buffer in 25 µL final volume. As DNA template, 120 ng and 300 ng were used to amplify the large and short target, respectively. The PCR parameters were, for the large mtDNA target, 2 min at 95°C, followed by 10 cycles of 10 s at 90°C, 20 s at 60°C and 5 min at 68°C, and then by 10 cycles of 10 s at 90°C, 20 s at 60°C and 5min, increasing 20 s per cycle, at 68°C, followed by a final step of 8 min at 68°C. For the short mtDNA target, the conditions were: 2 min at 99.9°C, followed by 25 cycles of 10 s at 95°C, 20 s at 60°C and 30s at 72°C, and a final step of 3 min at 72°C. Samples from the same individual chicks were always allocated in duplicate in the same plate, and with similar numbers of different experimental groups. We selected a sample with high levels of damage as a reference to inter-run normalization. Thus, the reference and 50% reference and no template (blank) control were included in duplicate in each microplate. PCR products were quantified by PicoGreen assay and corrected by blank fluorescence values. We optimized the amplification linearity that was considered acceptable if the 50% reference showed a 40-60% reduction of amplification signal. Duplicates were highly repeatable (large mtDNA target, r_{ICCC}= 0.971, P < 0.001; small mtDNA target, r_{ICCC}= 0.901, P < 0.001, n = 89). Relative DNA lesion frequencies were normalized to reference as previously described[11]. Briefly, we estimated the relative damage per DNA strand as the ratio of fluorescence values of large and small mtDNA target in each sample (RS) and in the reference (RR). Normalized mtDNA damage was

determined as –ln(RS/RR) and these values were highly repeatable when estimated for each replicate (r_{ICCC}= 0.899, P < 0.001).

Citrate synthase activity

Citrate synthase activity was measured in blood samples at age eight days. Citrate synthase is a mitochondrial matrix enzyme typically used as a marker of mitochondria abundance[12,13]. In a preliminary study of gull chicks, we found that the activities of citrate synthase and cytochrome c oxidase (Complex IV, a respiratory chain enzyme) were highly correlated (r=0.62, P<0.001, n = 45; unpublished data). Thus, citrate synthase activity may be a good proxy of the abundance of functional mitochondria. We followed the assays previously described in[13] with minor modifications. We first homogenized 100 mg of frozen blood cells in a sucrose homogenization buffer (250 mM, pH 7.4 at 4°C) in a 1.5 mL plastic tube on ice, and then the homogenate was centrifuged at 600g for 10min at 4°C. Three aliquots of supernatant were flash frozen in liquid nitrogen and stored at -80°C until analyzed. One aliquot was used to determine the sample protein content (Pierce BCA Protein Assay Kit; Thermo Scientific). We used a Synergy HT BioTek microplate reader to measure citrate synthase, adding 10 µL homogenate supernatant (0.6 µg proteins per µL), 110 mM Tris buffer (pH 8, 0.2 % (v/v) triton 100X), 100µM 5,5′-dithiobis(2-nitrobenzoic acid) and 310µM acetyl coenzyme A in each well. The reaction was initiated by adding 13 µL of 10 mM oxaloacetic acid. The change in absorbance at 412 nm during 3 min was monitored and the citrate synthase activity was expressed as the rate of production of thionitrobenzoic acid (nmol min^{-1} mg^{-1} of protein[13]; interassay CV= 4.06%). Citrate synthase activity was measured in triplicate (r_{ICCC}= 0.85, P < 0.001, n = 45).

Gene expression

The expression profiles of candidate genes were estimated based on relative quantification of mRNA transcripts, assayed by RT-qPCR using a StepOnePlus Real-Time PCR Systems (Applied Biosystems, Forest City, CA).

Blood samples in RNAlater from eight-day-old chicks were embedded in 200 µl of RiboZol (Amresco) and were homogenized using RNase-free pellet pestles (Sigma-Aldrich) until they were totally disaggregated. A volume of 800 µl of RiboZol was added to each homogenized sample to obtain a final volume of 1 ml per sample. The prepared samples were kept at -80 °C until the isolation of RNA to ensure the RNA integrity. Total RNA was isolated following the

RiboZol manufacturer's instructions. All samples were treated with DNase I to remove any contaminating DNA and the treated RNA was purified using the DNA-Free RNA kit (Zymo Research). The concentration and quality of RNA in each sample was quantified using a microplate spectrophotometer (Synergy HT with Take3, BioTek). Some samples (*n*=14) showed low amounts of RNA and were excluded from qPCR analyses. First-strand cDNAs were synthesized with qScript cDNA Synthesis Kit (Quanta Biosciences) using 500 ng of total RNA. The cDNA was stored at -20°C until real-time quantitative PCR (RT-qPCR) analysis.

Beta-actin gene (*ACTB*) has been identified as stable housekeeping genes in a previous study of yellow-legged gull[14], so it was used as reference gene. Gene-specific primers (Supplementary Table S5) were designed using sequence information obtained from previously published sequences for *ACTB*, *KIT*, *CASP7*, and *SIRT1* genes in the yellow-legged gull ([14]; Supplementary Table S5). We also designed primers for *NRF1* (Supplementary Table S5) using the available sequences in Charadriiformes *(Charadrius vociferous:* XM_009882988.1 and XM_009882989.1; *Calidris pugnax*: XM_014945424.1, XM_014945425.1 XM_014945426.1). All selected primers produced a single amplicon of expected size as confirmed by melt curve and electrophoresis analyses. Primers were synthesized by Sigma-Aldrich Química (Madrid, Spain).

The efficiency of each amplicon was estimated from the slopes of the amplification curves for each qPCR reaction and averaged for each gene using LinRegPCR software[6,15]. All selected primers showed an estimated efficiency between 1.890 and 1.933 (ACTB*:* 1.896 [range, 1.886-1.901], KIT*:* 1.933 [1.915-1.944], CASP7*:* 1.890 [1.885-1.892], SIRT1*:* 1.928 [1.918-1.933], NRF1*:* 1.909 [1.903-1.917]. The level of expression was measured in a 20 µl reaction volume, containing 0.8 µl of each primer (10 µM), 10 µl of Luminaris Color HiGreen qPCR Master Mix, high ROX (Thermo Fisher Scientific) and 1 µl of sample. The cycling conditions were set at 95°C for 5 min, followed by 40 cycles of 95°C for 15 s and 60°C for 1 min. All reactions were performed in duplicate. Cq values and estimated counts (by the MCMC.qpcr package) were highly repeatable in all amplicons (r_{ICCC}>0.95 in all cases).

References

1 Brambilla, G., Fiori, M. & Archetti, L. I. Evaluation of the oxidative stress in growing pigs by microplate assays. *Journal of Veterinary Medicine Series A* **48**, 33-38 (2001).

2 Kim, S. Y., Noguera, J. C., Tato, A. & Velando, A. Vitamins, stress and growth: the availability of antioxidants in early life influences the expression of cryptic genetic variation. *Journal of Evolutionary Biology* **26**, 1341-1352 (2013).

3 Alberti, A., Bolognini, L., Macciantelli, D. & Caratelli, M. The radical cation of N,N-diethyl-para-phenylendiamine: A possible indicator of oxidative stress in biological samples. *Research on Chemical Intermediates* **26**, 253-267 (2000).

4 Noguera, J. C., Kim, S. Y., Velando, A. Family-transmitted stress in a wild bird. Proceedings of the National Academy of Sciences, 201706164 (2017).

5 Kim, S.-Y. & Velando, A. Antioxidants safeguard telomeres in bold chicks. *Biology Letters* **11**, 20150211 (2015).

6 Ruijter, J. M. *et al.* Amplification efficiency: linking baseline and bias in the analysis of quantitative PCR data. *Nucleic Acids Research* **37**, e45-e45 (2009).

7 Furda, A., Santos, J. H., Meyer, J. N. & Van Houten, B. in *Molecular Toxicology Protocols* (eds Phouthone Keohavong & Stephen G. Grant) 419-437 (Humana Press, 2014).

8 Sanders, L. H., Howlett, E. H., McCoy, J. & Greenamyre, J. T. Mitochondrial DNA damage as a peripheral biomarker for mitochondrial toxin exposure in rats. *Toxicological Sciences* **142**, 395-402 (2014).

9 Santos, J. H., Meyer, J. N., Mandavilli, B. S. & Van Houten, B. in *DNA Repair Protocols: Mammalian Systems* (ed Daryl S. Henderson) 183-199 (Humana Press, 2006).

10 Hunter, S. E., Jung, D., Di Giulio, R. T. & Meyer, J. N. The QPCR assay for analysis of mitochondrial DNA damage, repair, and relative copy number. *Methods* **51**, 444-451 (2010).

11 Furda, A. M., Bess, A. S., Meyer, J. N. & Van Houten, B. in *DNA Repair Protocols* (ed Lotte Bjergbæk) 111-132 (Humana Press, 2012).

12 Larsen, S. *et al.* Biomarkers of mitochondrial content in skeletal muscle of healthy young human subjects. *The Journal of Physiology* **590**, 3349-3360 (2012).

13 Spinazzi, M., Casarin, A., Pertegato, V., Salviati, L. & Angelini, C. Assessment of mitochondrial respiratory chain enzymatic activities on tissues and cultured cells. *Nature Protocols* **7**, 1235 (2012).

14 Diaz-Real, J., Kim, S. Y. & Velando, A. Plumage colour and the expression of stress-related genes in gull chicks. *Journal of Avian Biology* **48**, 1216-1225 (2017).

15 Robledo, D. *et al.* Analysis of qPCR reference gene stability determination methods and a practical approach for efficiency calculation on a turbot (*Scophthalmus maximus*) gonad dataset. *BMC Genomics* **15**, 648 (2014).
